# Therapeutic application of T regulatory cells in composite tissue allotransplantation

**DOI:** 10.1186/s12967-017-1322-5

**Published:** 2017-10-26

**Authors:** Jeong-Hee Yang, Seok-Chan Eun

**Affiliations:** Department of Plastic and Reconstructive Surgery, Composite Tissue Allotransplantation Immunology Laboratory, Seoul National University College of Medicine, Seoul National University Bundang Hospital, Seongnam, South Korea

**Keywords:** Composite tissue allotransplantation, Immune rejection, Skin antigenicity, T regulatory cell, Immune tolerance, Immunosuppressive drug, Cell therapy

## Abstract

With growing number of cases in recent years, composite tissue allotransplantation (CTA) has been improving the quality of life of patient who seeks reconstruction and repair of damaged tissues. Composite tissue allografts are heterogeneous. They are composed of a variety of tissue types, including skin, muscle, vessel, bone, bone marrow, lymph nodes, nerve, and tendon. As a primary target of CTA, skin has high antigenicity with a rich repertoire of resident cells that play pivotal roles in immune surveillance. In this regard, understanding the molecular mechanisms involved in immune rejection in the skin would be essential to achieve successful CTA. Although scientific evidence has proved the necessity of immunosuppressive drugs to prevent rejection of allotransplanted tissues, there remains a lingering dilemma due to the lack of specificity of targeted immunosuppression and risks of side effects. A cumulative body of evidence has demonstrated T regulatory (Treg) cells have critical roles in induction of immune tolerance and immune homeostasis in preclinical and clinical studies. Presently, controlling immune susceptible characteristics of CTA with adoptive transfer of Treg cells is being considered promising and it has drawn great interests. This updated review will focus on a dominant form of Treg cells expressing CD4^+^CD25^+^ surface molecules and a forkhead box P3 transcription factor with immune tolerant and immune homeostasis activities. For future application of Treg cells as therapeutics in CTA, molecular and cellular characteristics of CTA and immune rejection, Treg cell development and phenotypes, Treg cell plasticity and stability, immune tolerant functions of Treg cells in CTA in preclinical studies, and protocols for therapeutic application of Treg cells in clinical settings are addressed in this review. Collectively, Treg cell therapy in CTA seems feasible with promising perspectives. However, the extreme high immunogenicity of CTA warrants caution.

## Background

Since the first successful human hand transplantation performed in 1998 [1] and human face transplantation performed in 2005 [2], composite tissue allotransplantation (CTA) for reconstruction of damaged or defected tissues has been rapidly emerging in the last two decades. Up to date, over 100 cases of hand transplantation and more than 30 cases of partial or full facial transplantation have been conducted worldwide [3, 4]. Advancements in CTA have been improving the quality of life of patient who has damaged tissues such as massive burns, cancer resections, congenital malformations, and accident-related traumas. Composite tissue allografts are heterogeneous. They are composed of various tissue types, including skin, muscle, vessel, bone, bone marrow, lymph nodes, nerve, tendon, and different transplanted elements with different immunogenic characteristics [5–7]. Skin, the primary target of CTA, has a rich repertoire of resident cells that play pivotal roles in immune surveillance. In this regard, understanding the molecular mechanism involved in immune rejection in the skin would be essential to successful CTA. Although there is scientific evidence demonstrating that the necessity of immunosuppressive drugs to prevent rejection of the transplanted tissues, 85–90% of hand transplant patients have experienced at least one episode of acute skin rejection with conventional immunosuppressive protocol, contrasts to the rejection rate of less than 10% in organ transplantation [8, 9]. There is a lingering dilemma in CTA because of limited responsiveness due to the lack of specificity and efficacy of targeted immunosuppressive drugs, which also have side effects such as risks of infection, cancer development, metabolic toxicity, and hypertension [10]. Maintaining immunosuppressive drugs on CTA would be challenging. Thus, there have been efforts to develop Treg cells with donor antigen-specificity to avoid harm to the body’s immune system.

Treg cells expressing CD4^+^CD25^+^Foxp3^+^ are a dominant form of T regulatory cell. The immune tolerant role of naturally arising Treg cells in autoimmune diseases and their effects on prolonged allograft survival in CTA with transplant rejection prevention function have been well documented in preclinical model studies [11, 12]. Moreover, several studies have demonstrated that Treg cells participate in allotransplantation tolerance across major histocompatibility complex (MHC) barriers, thus, broadening the applicability of Treg cells in CTA [13, 14]. Mechanisms involved in the immune tolerant function of Treg cells targeting inhibition of T effector (Teff) cells can be described as follows: (1) suppression of dendritic cells (DCs) maturation and production of indoleamine 2,3-dioxygense; (2) production of granzyme A/B and perforin that leads to lysis of Teff cells; (3) deprivation of IL-2 via high affinity CD25 (IL-2Rα); (4) release of inhibitory cytokines such as TGF-β, IL-10, and IL-35; (5) production of cAMP metabolites with disruption of metabolomics in Teff cells [15]. In addition, immune regulatory modalities of Treg cells combined with immunosuppressive drugs have offered options of Treg cell therapy in CTA [16].

In this updated review on therapeutic application of Treg cells in CTA, molecular and cellular characteristics of CTA and immune rejection, Treg cell development and phenotypes, Treg cell plasticity and stability, immune tolerant functions of Treg cells in CTA in preclinical studies, and protocols for therapeutic application of Treg cells in clinical settings are addressed. Collectively, Treg cell therapy in CTA seems feasible with promising perspectives. Managing skin antigenicity would be essential to achieve successful CTA and allotransplantation in general.

## Characteristics of CTA and immune rejection

High rate of acute graft rejection is one main characteristic of CTA which is composed of diverse layers of tissues [7]. In particular, the antigenicity of skin has been amply demonstrated by multiple rejection episodes in clinical hand transplant patients [17]. It has been reported a rich repertoire of skin-resident immune cells play pivotal roles in immune surveillance [18, 19]. Examination of early human skin allograft has shown that endothelial cells of microvasculature are the critical targets of CD4^+^ and CD8^+^ T lymphocytes of recipients in first-set vascularized skin allograft rejection, resulting in extensive microvascular damage [20, 21]. By contrast, in facial allografts, analysis of acute rejection has shown that donor lymphocytes are major sources of immune rejection in the rejected area [22]. In addition, abundant skin-resident T cells of donor origin are found to be associated with vascular, pilosebaceous, and epidermal sites of injury [22]. Considering that skin is the primary target of immune rejection in CTA, it seems reasonable to pay attention to the skin resident cells participating in immune surveillance due to multiple episodes of skin allografts rejection. Skin explants rejection studies have revealed that Langerhans cells with expression of unregulated MHC class II antigens are migrated out of the skin, and their maturation proceeded by local inflammatory cytokines eventually leads to rejection [23]. Microscopic observation of skin allografts has revealed that the dermis appears to be spared while the epithelium is destroyed in transplantation rejection [24]. These results indicate that elucidating molecular and cellular characteristics of skin and resident cells might help us understand the episodes of skin allograft rejection. A variety of immune cells such as Langerhans cells, dermal DCs, macrophages, mast cells, and diverse T cell receptor (TCR) repertoire of T cells [T effector memory (T_EM_) cells of CD4 and CD8, Treg cells, and tissue resident memory T cells (T_RM_)] are located in normal skin [25]. It has been estimated that 98% of cutaneous lymphocyte antigen (CLA) T_EM_ cells reside in the normal skin [26]. These cells can cause rapid and sensitive immune reactions in allografts with expression of CD69, CD103, and CLA. A large pool of memory T cells in the skin can be distinguished from memory T cells in the blood by differential pattern of gene expression. They can mediate immune reactions without recruiting T cells from the blood [27]. Signs of rejection in CTA patients with administration of immunosuppressive drug are sharply contrasted to those of kidney transplantation patients whose immune rejection is fairly controlled [10, 19, 20, 28]. Interestingly, split tolerance and hierarchy of antigenicity as characteristics of CTA have been observed in animal studies. A MHC-I mismatched tolerant kidney of swine model has demonstrated that CTA is accepted for a long time with the exception of the epidermis [29]. Exposure of tolerant animals to second donor-matched kidneys prior to CTA has increased the longevity of CTA epidermis [29]. In a limb allograft model, individual tissue graft of the muscle or skin has shown greater cellular and humoral immune rejection rates than total limb grafts [30]. These results highlight the importance of immune modulation of skin antigenicity for successful CTA.

The development of modern immunosuppressive drugs has prevented rejection of allografts. It had opened a new era of CTA [7]. Following the first successful human kidney transplantation between twin brothers in 1954 [31], azathioprine and prednisolone have been introduced to renal transplantation. Polyclonal anti-thymocyte globulin (ATG) preparations became available in the 1970s [32]. In the 1980s, cyclosporine became the first single drug that could control graft rejection [33]. Trials that demonstrate the benefit of cyclosporine in kidney recipients compared to azathioprine and steroids have assured the use of cyclosporine in solid-organ transplantation [33]. Subsequently, a combination of immunosuppressive regimen given at the time of transplantation (induction therapy) and during maintenance phase is used conventionally in CTA [34]. Although immunosuppressive drugs can almost completely prevent acute rejection rooted in the induction of immune tolerance during transplantation, chronic rejection of organ allografts still remains a problem. In addition, the use of non-specific immunosuppressive drugs has various side effects, including the development of multiple malignancies [10]. In general, patients with CTA face rather tough decision over organ transplantation when the issue is aesthetic than life-threatening.

The concept of induced tolerance to allografts arose from the pioneering work of Nobel laureate Sir Peter Medawar and colleagues. They have suggested that acquired tolerance is due to specific failure of the host’s immunological response and that the antigenic properties of homografts (allografts) are not altered by residence in a tolerant host as the host itself retains the power of passively acquired immunity directed against a homograft (allograft) [35]. In terms of the concept of induction of tolerance to the host to prevent allograft rejection, Treg cells are likely to fit such concept. It has been found that CTA is well accepted up to 6 years after transplantation in the presence of Treg cells with expression of immune suppressive cytokines, such as interleukin-10 (IL-10) or transforming growth factor-β (TGF-β) [36]. The involvement of Treg cells in inhibition of transplant arteriosclerosis combined with rapamycin treatment known to expand Treg cells suggests that adoptive transfer of Treg cells in induction of immune tolerance in CTA is feasible [37]. Thus, preventing immune responses to foreign cells and tissues by Treg cells may offer a new way to minimize reliance on non-specific immunosuppressive drug. This could ultimately allow patients to be completely withdrawn from drug-based immunosuppression.

## Treg cell development and phenotypes

Treg cells are associated with a variety of immune related reactions, including autoimmune disease, transplantation, allergy, infection, and cancer. A dominant form of the subset is CD4^+^CD25^+^Foxp3^+^ Teg cells. Transcription factor Foxp3 is known as a mast regulator central to the development of Treg cells [38, 39]. Mutations in Foxp3 gene is directly linked to severe multi-organ autoimmune diseases, including immune dysregulation polyendocrinopathy enteropathy X-linked syndrome (IPEX) in human and scurfy mice [40, 41]. To simplify the nomenclature, Treg cells are classified into three types: thymic Treg (tTreg) cells induced in the thymus, peripherally induced Treg (pTreg) cells, and in vitro induced Treg (iTreg) cells [42]. In the thymus, tTreg cells are developed from bone marrow derived precursor cells in the cortex and medullary region via the following two steps. The first step involves the development of a Foxp3^−^CD25^hi^ T cell intermediate in TCR dependent manner. The second step involves the development of Foxp3^hi^ T cells under the influence of IL-2 independent of TCR [43–45]. Most self-antigen reactive T cells are deleted centrally during T cell development while tTreg cells are exported to the periphery to maintain immune tolerance and immune homeostasis. Although signals for the generation and function of pTreg cells are less clear, pTreg cells are induced in the periphery by antigen presentation in non-immunogenic, inflammatory, and non-inflammatory conditions [46, 47]. In mice subjected to neonatal thymectomy, analyses of adoptive T cell transfer into lymphopenic hosts have led to the identification of a specialized subset of pTreg cells capable of suppressing autoimmunity and immune suppressive function under inflammation. Especially, pTreg cells function in immune tolerance is well manifested at the mucosal site [48, 49].

During development, a population of FoxP3^−^CD25^hi^ T cell intermediates derived from thymic precursor T cells is precommitted to differentiate into Foxp3^+^ Treg cells upon TCR stimulation [50, 51]. On this line, periphery T cells termed recent thymic emigrants (RTE) are observed to be exclusive precursor cells of pTregs. They can be developed into Foxp3^bright^CD25^high^ Treg cell phenotypes in the human and mice [52]. Interestingly, a study has shown that pTreg cells can recirculate to the thymus and suppress the development of their precursors [53]. This indicates that development of tTreg and pTreg cells is tightly co-regulated and balanced. Interestingly, tTreg cell population renders naïve T cells to be tolerant, thus, creating ‘infectious tolerance’ by generating pTreg cells through delivery of TGF-β expressed on tTreg cells in transplantation [54, 55]. For the generation of iTreg cells, in vitro animal model studies have found that mice naive CD4^+^CD25^−^ T cells can be induced to express CD25^+^Foxp3^+^ in presence of IL-2 and TGF-β [56, 57]. Based on such observation in mice, initially, it has been assumed that the generation of human iTregs could be recapitulated under the condition used for mice. Unexpectedly, human iTreg cells expressing Foxp3 in presence of TGF-β failed to suppress proliferation of responder CD4^+^Foxp3^−^ T cells [58]. When re-stimulated with PMA or ionomycin, majority of Foxp3^+^ iTreg cells produced IL-2 and/or IFN-γ [58]. These results suggest that induction of iTreg cells in mice and human system does not seem to be the same. However, another group has successfully induced immunosuppressive human iTreg cells from peripheral blood mononuclear cells (PBMCs) in healthy adults by using retinoic acid (RA), IL-2 and TGF-β [59]. Moreover, a recent study has demonstrated that induction of highly suppressive iTreg cells from human umbilical cord blood treated with TGF-β and all-trans RA is mediated by the expression of micro RNAs (miRNAs) which can selectively repress genes related to Th17 differentiation [60]. Thus, in vitro expansion of Treg cells to induce immune tolerance therapeutically seems highly promising.

In lineage commitment of Treg cells, an important distinction between tTreg cells and extrathymic Treg cells is that a strong TCR signaling with CD28 co-stimulation just below the threshold for negative selection is necessary for tTreg cells. Extrathymic conversion of naive T cells into Tregs is inhibited by CD28 costimulation, although such costimulation is critical for survival of pTreg cells which favor weak TCR stimulation [61]. Peptide-MHC ligand potency, density, and duration of TCR interactions are factors that might induce pTreg cells [62]. Under TCR signaling, it has been found that nuclear factor-kappa B (NF-kB) pathway leading to c-Rel is correlated with the expression of Foxp3 as a defect in connecting TCR to NF-kB that curtails Treg cells differentiation [63], indicating that the TCR-NF-kB-c-Rel axis is the main pathway for Foxp3^+^Treg cell development. In addition, nuclear factor of activated T cell (NFAT)-Foxp3 complex can mediate expression of CTLA-4 and CD25, markers for Treg cells [64]. It is notable that the activity of Foxo-1 transcription factor needs to be repressed by Akt kinase during TCR stimulation [65].

Phenotypic standard markers of Treg cells are still controversial. However, upregulated expression of Helios and Nrp-1 in tTreg cells [47] and expression of Helios with high proportion of IL-10, Ebi31, and CTLA-4 in tTreg cells suggest that tTreg cells are distinguishable from pTreg cells [66]. These results are challenged by the observation that there are no differences in expression levels of Helios and Nrp-1 between tTregs and pTreg cells in both mice and human systems [67, 68]. In the human system, identification of phenotypic Treg cell marker is more difficult because of its heterogeneity and stability. Not surprisingly, functional heterogeneity within human FOXP3^+^ Treg cells has been observed in single cell analysis [69]. In that study, most human primary Foxp3^+^ clones have potent suppressive abilities while around 25–30% of Foxp3^+^ clones are lack of suppressive capacity against conventional T (Tconv) responders. Among population of Helios^+^Foxp3^+^ Treg cells enriched for suppressive clones, Helios^−^Foxp3^+^ Treg subpopulation is found to harbor nonsuppressive clones with production of pro-inflammatory cytokines such as IFN-r, IL-2, and IL-17 [70]. This implies that Helios might be a solid phenotypic marker for Treg cells. GITR, CD39, TNFR2, HLA-DR, CTLA4, GARP, and LAP are expressed in human Treg cells [71]. They could be considered as presumptive phenotypic markers. However, these molecules are also expressed on activated Tconv cells under inflammatory condition [72]. Nevertheless, CD4^+^CD25^+^Foxp3^+^Treg cells are the best known and the most dominant subset of Treg cells participating in immune tolerance reaction and immune homeostasis so far. Other types of T cells with regulatory activity but not expressing Foxp3 include type 1 regulatory cells (Tr1 cells), CD4^+^CD25^+^LAG3^+^ T cell, Th3, iTr35, CD8^+^T, CD4^−^CD8^−^ double negative T cells, NK T cells, and γδ T cells [73, 74]. A range of cytokine expression and chemokine receptors that can promote differential tissue localization have been observed in these cells. Particularly, immune tolerant activities of Tr1 cells that can secret IL-10 have drawn a lot interest in future clinical application [75]. In summary, identification of reliable phenotypic markers for Treg cells may pave a way for therapeutic application.

## Treg cell plasticity and stability

The application of immunosuppressive Treg cells for protection against allograft rejection remains rather elusive due to the lack of specific markers. Although Foxp3 is considered as gold standard for Treg cells and extremely resilient with the same immunosuppressive function in mice [45, 76], it is notable that human Treg cells are unstable under various inflammatory conditions [72]. In addition, several studies have confirmed that Tconv cells under inflammatory condition express Foxp3 as well [58, 71, 77]. In this regard, our endeavors for application of bona fide Treg cells in CTA seem rather promiscuous due to their plasticity and lack of stability. Studies have shown that Treg cells can lose immune tolerant functions by diverse mechanisms, including co-stimulatory pathways, degradation of Foxp3, epigenetic regulation, and cytokine interaction [78, 79]. It is currently unclear whether any single pathway can regulate Treg cells exclusively. It is assumed that co-stimulatory pathways might differentially regulate Treg and Tconv cells. Despite shared TCR sequences between Treg and Tconv cells, the lineage of Treg cells with particular balance and intensity of TCR signals are different from Tconv cells [44, 80]. In support of unique TCR signaling in Treg cells, non-overlapping unique CDR3 sequences between tTreg and Tconv cells have been elucidated by sequencing analysis [47]. In vitro expanded mice iTreg cells with combined treatment with anti-CD3, anti-CD28, and IL-2 have well-maintained functional activities [81]. However, it has been found that but human Treg cells repeatedly stimulated in vitro with anti-CD3 and anti-CD28 antibodies will lose Foxp3 expression, resulting in the generation of pathogenic memory T cells [82, 83]. The stability of Treg cells can also be lost by ubiquitination of Foxp3, leading to degradation Foxp3 via signal transducer and activator of transcription 3 (STAT3) signaling. In a STAT5 limited environment, the mTOR/AKT/PI3K pathway and PDL-1can inhibit Foxp3 expression whereas Foxo1 and Wnt signaling can enhance Foxp3 expression, thus inducing immune suppressive function of Treg cells [78, 84]. Epigenetic regulation is another contributing factor to the stability of Treg cells. Epigenetic hypomethylation in Treg-specific demethylated region (TSDR) is crucial to maintaining high Foxp3 expression. Methylation at this site has been reported to be different between Treg cells and CD4^+^Tconv cells [85]. Likewise, Treg cells generated in vivo are more strongly demethylated in the Foxp3 locus. They are able to maintain their stability compared to Treg cells generated in vitro [86]. On this line, increased CpG methylation in a conserved region of the Foxp3 gene in CD4^+^CD25^+^CD127^low^ Treg cells is found to be correlated with the loss of Foxp3 expression and increased production of pro-inflammatory cytokines [82]. Concerning the convertibility of Treg cells, expression of surface markers such as CD45RA or CD127 might be able to define the fate of Treg cells. In a recent study on patients with kidney transplantation tolerance, hypomethylation has occurred on Foxp3 TSDR in CD45RA^−^Foxp3^hi^ memory Treg cells [87], suggesting that population of CD45RA^−^Foxp3^hi^ memory Treg cells might produce favorable outcomes in CTA. However, caution is needed as Foxp3 expression does not always guarantee suppressive function of Treg cells. For example, when naive human CD4^+^CD25^−^CD127^+^CD45RA^+^ T cells from PBMCs are induced to express high levels of Foxp3 in the presence of IL-2 and TGF-β, the production of IFN-γ and IL-2 inflammatory cytokines has failed to exert immunosuppressive Treg cells although Foxp3 expression in these cells is stable [58]. Moreover, variations and inconsistent Foxp3 expression have been observed in T cells expressing high levels of CD25 even in healthy individuals in addition to high levels of CD25 expression in Tconv cells under inflammatory condition. Thus, plasticity and stability of human Treg cells should be considered with great importance, particularly, under inflammatory condition associated autoimmune diseases.

The influence of inflammatory condition on the stability of Treg cells is well manifested by the reversible conversion between Treg cells and T17 cells based on observation that Treg cells treated with IL-6 and IL-1 express IL-17 [88]. Recent studies have indicated that IL-15 can fine tune the balance between the expression of Foxp3 and IL-17 mediated by RAR-related orphan receptor γt, a Th17-specific transcription factor [89, 90]. RA can inhibit Th17 polarization and enhance Foxp3 expression through STAT-3/STAT-5 independent signaling pathway [91]. A recent study has revealed that induction of Treg cells by treatment with RA and TGF-β is mediated by miRNAs which can exclusively suppress genes related to differentiation of Th17 cells [60]. Collectively, dynamic plasticity and stability of Treg cells may lead to heterogeneity of Foxp3^+^ Treg cell subsets and diversity of responsiveness in immune tolerance and immune homeostasis. Therefore, they should be considered carefully in CTA.

## Immune tolerant function of Treg cells in CTA

Survival of allografts and prevention of chronic rejection are two main goals in CTA.

However, vigorous immune response of Teff cells triggered by alloantigen is the main obstacle to achieve these goals. There are three pathways of alloantigen recognition by Teff cells depending on the pattern of antigen presentation: (1) in a direct pathway, recipient TCR recognizes donor antigen bound to donor MHC molecule presented by donor APCs; (2) in an indirect pathway, recipient TCR recognizes donor antigen bound to recipient MHC molecule presented by recipient APCs; (3) in a semi-direct pathway, recipient TCR recognizes donor antigen bound to donor MHC molecule presented by recipient APCs [92]. It has been suggested that the direct pathway participates in acute graft allorejection whereas the indirect pathway participates in chronic graft allorejection with production of alloantibodies [93]. However, the role of the semi-direct pathway in allotransplantation has not been clearly addressed yet [93]. Treg cell-mediated immune tolerance and tissue damage control in CTA rely on the inhibition of Teff cells that can recognize alloantigens. For Treg cell therapy in CTA, the suppression of Teff cells with adoptive Treg cell transfer might be hampered by a low number of natural Treg cells and anergic phenotypes, thus losing their immunosuppressive function during in vitro expansion. Large scale in vitro expansion of mice and human polyclonal CD4^+^CD25^+^Treg cells has revealed that their immunosuppressive functions are maintained [81, 94], supporting that their clinical application in CTA might be feasible.

Cumulative evidence has demonstrated that effective immune tolerant Treg cells can function on CTA in preclinical studies [11, 95]. In early days, studies on histo-incompatible thymic epithelium grafts have suggested the immune tolerant potential of Treg cells in allotransplantation [96–98]. Depletion of CD25^+^ cells at the time of bone marrow transplantation and blocking CTLA-4 have abrogated the development of tolerance [99], which suggesting an essential role of IL-2 and CTLA4 in immune tolerant Treg cells function. Interestingly, a subset of Treg cells expressing low levels of CD127 are 5 times more potent in tolerance induction in an animal model of transplanted arteriosclerosis by inhibition of Teff cell function and graft infiltrations [100]. Since the phenomenon of arteriosclerosis in transplantation is considered as a model of chronic transplantation rejection, this result indicates that Treg cells might have potency in long term immune tolerance. Accordingly, Treg cells sorted for the expression of CD4^+^CD25^+^CD127^low^ have displayed potent immune suppressive capability compared to conventional CD25^hi^CD4^+^ Treg cells. The contribution of host Treg cell population to induction of immune tolerance has been observed in hepatic allografts model in human and mice systems compared to transplantation rejected model and bone marrow allografts [101, 102]. Therefore, boosting the number of recipient Treg cells might have favorable outcomes in CTA. On the other hand, rapid development of lethal graft-versus-host disease (GVHD) has been observed after administration of cultured CD4^+^CD25^+^ T cells with an equal number of CD4^+^ T cells or CD25 depleted whole T cells [103]. In addition, the level of Treg cells in peripheral blood does not predict outcomes of transplantation in a nonhuman primate model [104].

Although application of Treg cells in induction of immune tolerance could be an advantage in CTA by minimizing or replacing non-specific immunosuppressive drugs, concerns have been raised about the possibility that the use of naturally occurring polyclonal Treg cells might cause systemic non-specific immunosuppression. In this regard, generation of antigen-specific Treg cells that can selectively induce immune suppression against donor antigen is highly encouraged. Such cells have demonstrated high efficacy [81, 105, 106]. Treg cells with indirect allospecificity for the A2 peptide with autologous DCs have shown potent antigen-specific suppression of skin allografts than polyclonal Tregs cells in the human and mice systems [107, 108]. Treg cells exposed to defined antigens can prevent the rejection of fully allogeneic skin grafts. Mice receiving skin allografts have shown expanded Treg cells that are preferentially accumulated in graft-draining lymph nodes or within the graft, suggesting the efficacy of donor antigen-specificity in transplant tolerance [109]. It has been reported that co-culture of naive CD4^+^ T cells and allogeneic antigen presenting cells (APCs) with TGF-β, RA and IL-2 can induce alloantigen-specific Treg cells with immunosuppressive function in skin allografts [110]. In stably engrafted skin transplants, introduction of Treg cells into mixed lymphocyte reaction had led to suppression of donor-directed T cell responses with increase of Foxp3^+^ cellular infiltrates [36, 111]. In addition, functional roles of surface molecules expressed on Treg cells have been addressed. It has been reported that Treg cells generated by exposure to an alloantigen in the peripheral blood that can prevent allograft rejection are mediated by CTLA-4 and IL-10-dependent pathways [112]. The clinical potential of infused Tregs cells and their subsequent prevention of acute and chronic allograft rejection have been investigated as follows. Treg cells specific for direct presentation of donor-antigens have failed to prevent chronic allograft rejection. However, Treg cells specific for indirect presentation of donor-antigens fully prevented chronic allograft rejection [105]. Treg cells with indirect allospecificity given by transduction of TCR genes that can confer specificity for MHC class II molecules have led to substantially greater efficacy in transplantation tolerance [113]. These findings implicate that overcoming immunological barriers would be crucial to have efficacious immune tolerance in allotransplantation. Application of donor antigen-specific Treg cells has proved its efficacy in CTA with antigen-specific immune suppression.

### Effect of skin resident cells on Treg cells

CTA is a complex process that involves coordinated innate and adaptive immune responses with multiple mechanisms. Skin is the primary target of CTA. It has extreme antigenicity compared to organ transplantation [114]. High levels of alloreactive memory T cells in the skin of sensitized recipient are formidable barriers to allotransplantation tolerance in nonprimates and human [115]. Accumulating evidence has indicated that direct modulation of Treg cells by skin resident cells, including DCs, memory Treg cells, macrophages, mast cells, and dermal cells may contribute to successful CTA (Table 1).

**Table 1:** Skin-resident cells interacting with Treg cells

Cell type	Action	References
Langerhans cells	Activate and proliferate Treg cells in resting state; limit activation of Treg cells in presence of pathogen	[116]
	Induce Treg cells favoring flora tolerance with limited antigens presentation	[117]
	Induce Treg cells by secretion of IL-10 and TGF-β	[118]
Memory Treg cells	Localize to hair follicles Non-migratory and non-responsive in normal skin Proliferate and produce IL-17 in inflamed skin	[119]
	Are activated, proliferated and differentiated into potent suppressor Attenuate autoimmune reactions in tissues upon repeated responses to antigens	[120]
Macrophages	Promote expression of the chemokine CCL22 Induce migration and activation of Treg cells	[121]
	Express M2-like TIM-4^hi^CD169^+^ Act immunoregulatory function and promote engraftment of cardiac allografts	[122]
Mast cells	Act as intermediate at Treg cells dependent allograft tolerance via IL-9	[123]
	Counteract Treg cell function through IL-6 and OX40/OX40L axis toward Th17 cell differentiation	[124]
Dermal dendritic cells	Are capable of antigen capture and presentation to CD4^+^ T cells and Treg cells generation	[125]
Dermal regulatory cells	Induce Treg cells through PD-1 engagement with expression of ABCB5^+^ molecules	[126]
Dermal fibroblasts	Induce proliferation of natural Treg cells with IL-15	[127]
Dermal stromal cells	Express CD90^+^ and induce Tregs cells	[128]

*CCL* chemokine ligand, *TIM* T cell immunoglobulin mucin, *ABCB5* ATP binding cassette subfamily B member 5

The function of DCs is notable in that deletion of Langerhans cells and dermal DCs will reduce immune tolerance. Therefore, their combined application with Treg cells seems encouraging [129, 130]. Previously, our lab has reported that tolerogenic DCs can prolong hind limb allografts survival when they are co-treated with FK506 [131]. Interestingly, DCs interacting with Treg cells in the skin are twice prevalent compared to those in peripheral blood [9]. Unconventional NK T cells can rapidly produce pro-inflammatory or anti-inflammatory cytokines in response to their cognate glycolipids antigens presented on CD1 molecules [132]. They are most frequently found in the liver (30–50%). However, their presence in the skin is not well reported. It has been reported that human skin NK T cells have 1.72–33% of cellular infiltrates in allergic contact dermatitis [133]. They produce IL-4 and IL-10 that can induce tolerogenic DCs and lead to expansion of Treg cells [134]. In addition, changes in expression of negative costimulatory receptors and anti-inflammatory cytokines by Treg cells in an IL-4-dependent manner can be promoted by NK T cells, resulting in tolerance to bone marrow and organ grafts [135]. In GVHD mice, bone marrow NK T cells can inhibit the acute lethal immune response by augmenting proliferation of donor-derived Treg cells in an IL-4-dependent manner [136, 137]. This suggests that NK T cells can induce immune tolerance. However, NK cell function in induction of immune tolerance does not seem supportive in which CD28-mediated conversion of CD4^+^CD25^−^ T lymphocytes into CD4^+^CD25^+^ Treg cells is inhibited by the release of IFN-γ [138]. More convincingly, direct lysis of activated Treg cells in response to microbial antigen is NKG2D- and NKp46-dependent, suggesting that NK cells have inhibitory effect on immune tolerance [139]. The positive role of APCs including macrophages, DCs, and B cells in CTA is also highly possible based on following findings. Studies on the regulatory role of macrophages have revealed that tacrolimus can contribute to graft survival and kidney transplantation without having deleterious effects [140]. Moreover, induction of Treg cells with direct allospecificity by tolerogenic DCs to prevent transplantation rejection is encouraging [141]. However, the role of B cells on allotrasplantation is unclear with positive and sometimes negative function. Studies have shown that B cells can produce IL-10 during inflammation and organ transplantation and cause the conversion from Tconv cells to Tr1 cells, thus preventing transplantation rejection [142, 143]. The function of B cells in expanding Treg cells with the requirement of TGF-β in signaling through TCR and CD28 has been reported [144]. In addition, when purified Treg cells are stimulated by CD40L-activated allogeneic B cells and expanded ex vivo with IL-2, greater protection against skin damage has been demonstrated in a humanized mouse model [145]. On the other hand, a contradictory result has been shown in the patient with the first human full face transplantation [146]. Class II-donor specific antibodies were developed at 90 months after transplantation with deposition of C4d in demal vessels, followed by skin rejection [147]. This suggests that B cells play a dual role (immune induction and immune tolerance) in transplantation regulation. Evidently, these findings suggest that innate or adaptive immune cells in the skin are important immune modulators. They may reinforce the feasibility of CTA in association with Tregs cells or independently. Further studies may clearly elucidate the path involved.

### Effect of immunosuppressive drugs on Treg cells

The outstanding efficacy of immune suppressive drugs for suppression of transplantation rejection has been remarkable and indispensable. However, nonspecific immunosuppression has side effects. To replace or minimize their side effects, attempts have been made vigorously. Ex vivo expanded Treg cells with tacrolimus, mycophenolate, and methylprednisolone, the three mostly used immunosuppressive drugs in transplantation clinic, have been investigated in preclinical model. Methylprednisolone reduces inflammatory reaction that may favor Treg cell function. Mycophenolate inhibits T and B cell proliferation without effect on Tregs. However, tacrolimus is detrimental on Treg cell function and survival [148]. The inhibitory effect of tacrolimus on the calcineurin pathway that lead to IL-2 production has been observed in the early phase of proliferation of Teff cells induced by IL-2 and in the late phase of survival of Tregs cells induced by IL-2 [47, 149, 150]. In addition, low dose IL-2 therapy that can selectively target Treg cells has shown positive outcomes in patients with Type 1 diabetes mellitus (DM) in clinical settings [151]. With tacrolimus, a population of human Treg cells expressing CD4^+^CD127^−/low^CD25^+^CD45RA^+^ Treg cells has stable TSDR demethylated FOXP3^+^ phenotype. They can maintain strong suppressive activity against CD4 Teff cell proliferation after expansion, suggesting that a defined population of Treg cells might be effective as therapeutic application by responding to tacrolimus [152]. It is noteworthy that rapamycin, an inhibitor of mTOR, can maintain the viability, function, and proliferative ability of adoptively transferred Treg cells in allotransplantation. It can induce Tregs cells in both healthy volunteers and patients with type I DM [153]. mTOR activation is essential for T cell commitment to Th1, Th2, and Th17 effector lineages. However, naive T cells preferentially differentiate into Treg cells in absence of mTOR [154]. Rapamycin can inhibit mTOR pathway as well as Smad7, a negative regulator of TGF-β signaling. Accordingly, adoptive transfer of a small number of alloantigen-specific Treg cells along with low dose of rapamycin has induced long-term survival of cardiac allografts in mice [155], thus, providing an informative regimen for Treg cell therapy with rapamycin. Taken together, immune tolerance function of Tregs in CTA can be synergized with immunosuppressive drugs such as tacrolimus and rapamycin.

## Therapeutic application of Treg cells in the clinic

### Ex vivo expansion of Treg cells for adoptive transfer

Isolation of blood Treg cells, expansion in ex vivo system, and reinfusion modalities have been attractive goals to increase the feasibility and efficacy of Treg cell therapy [148]. To have quality production of Treg cells for clinical use, Good Manufacturing Product (GMP) facilities are mandatory. They should fulfill safety concern and generate clinically relevant number of Treg cells with maximum purity that can abundantly express Foxp3 without secreting pro-inflammatory cytokines [156]. Although it is challenging, in vitro expansion protocols have been established by several groups for polyclonal Treg cells and antigen-specific Treg cells from peripheral blood and umbilical cord blood after apheresis, respectively [81, 94, 157, 158]. About 2–10% of CD4^+^ T cells are present in human Treg cells. However, only 0.25 × 10^9^ Treg cells among the total number of 13 × 10^9^ Treg cells are circulating in the blood [159]. To isolate Treg cells, CD4^+^CD25^+^ T cells are selected following the depletion of CD8^+^, CD14^+^, and CD19^+^ cells after depleting CD8^+^ T cells, monocytes, and B cells using antibody-coated magnetic-beads, respectively. A positive selection step is then used to enrich CD4^+^CD25^+^ T cells using a sub-saturating concentration of anti-CD4 antibody and anti-CD25 antibody to capture the CD4^+^ and CD25^bright^ fractions. In the preparation of CD4^+^CD25^+^Foxp3^+^ Treg cells, no consensus procedure has been set up yet. Up to date, the following four types of GMP-grade clinical Treg cells have been proposed depending on surface marker: CD4^+^CD25^+^ T cells, bona fide Treg cells expressing CD4^+^CD25^+^CD127^low/−^, naïve Treg cells expressing CD4^+^CD25^+^CD127^low/−^CD45RA^+^, and CD4^+^CD25^+^CD127^low/−^GARP^+^LAP^+^ [160]. Although human and murine Treg cells are anergic and unsuitable for large-scale expansion, studies have shown that supplementation of high-dose IL-2 in combination with TCR- and CD28-mediated stimulation is sufficient to promote their in vitro proliferation [94]. Up to date, most adoptive Treg cell therapy studies have used CD4^+^CD25^++^ or CD4^+^CD25^+^CD127^−^ Treg cells. It is possible that Treg cell subset appropriateness will vary depending on the application. In addition, it has been reported that CD4^+^CD25^high^CD45RA^+^ Treg cells are ideal as starting population for the generation of homogeneous and stable Treg cells [161]. In GVHD, large-scale expanded human iTreg cells have successfully manifested their immune suppressive functions [162], indicating that Treg cell therapy is promising in CTA.

### Production of donor antigen-specific Treg cells

As highlighted in preclinical model studies, antigen-specific Treg cells have higher potency in suppressing undesired immune responses compared to polyclonal Treg cells. To produce donor antigen-specific Treg cells, DCs, B cells, and PBMCs have been used [163]. To prepare allogenic DCs-specific Treg cells, CD4^+^CD25^+^CD127^low^ T cells derived from PBMCs in the recipient have been treated with CD1c^+^ dermal DCs derived from donor skin. After priming with DCs in mixed lymphocyte reaction and stimulation with CD3 and CD28 antibodies, enriched and expanded donor antigen-specific Treg cells with expression of CD69^+^CD71^+^ can be harvested [164]. For B cells, alloantigenicity can be obtained by stimulation with CD40L-activated allogeneic B cells from the donor or allogeneic human leukocyte antigen (HLA) [165]. Manufacturing and clinical trials of antigen-specific Treg cells therapy to induce immune tolerance in allotransplantation and autoimmune diseases are underway [160, 163].

Alternatively, antigen-specific Treg cell therapy using chimeric antigen receptor (CAR) expression system has generated interest. Human Treg cells engineered to create HLA-A2-specific CAR have prevented xenogeneic GVHD in mice model [166], suggesting promising perspectives. CAR-Treg-cell therapy targeting donor antigen with enhanced immunosuppressive activity has been demonstrated in autoimmune diseases. It has efficiently prevented autoimmune encephalitis (EAE), colitis, and immune responses to factor VIII in hemophilia A [167–169]. Another approach involves the engineering of TCR gene to induce tolerance in CTA in a rodent model. In this approach, CD4^+^CD25^+^ Treg cells with indirect allospecificity can be generated by retroviral transduction of TCR genes specific for allogeneic MHC class II. Induction of indefinite heart allograft survival with short-term systemic immunosuppression in partially MHC mismatched heart allografts combined with immunosuppressive drugs has been reported [113]. With the goal to manipulate TCR gene as therapeutics, RNA-interference and genome editing technique using transcription activator-like effector nucleases to increase efficacy and safety have been attempted [170]. These data collectively suggest that CAR-Treg cells and modifying TCR gene might be useful for CTA clinically in the future.

### Clinical trials of Treg cells

**Fig. 1:**
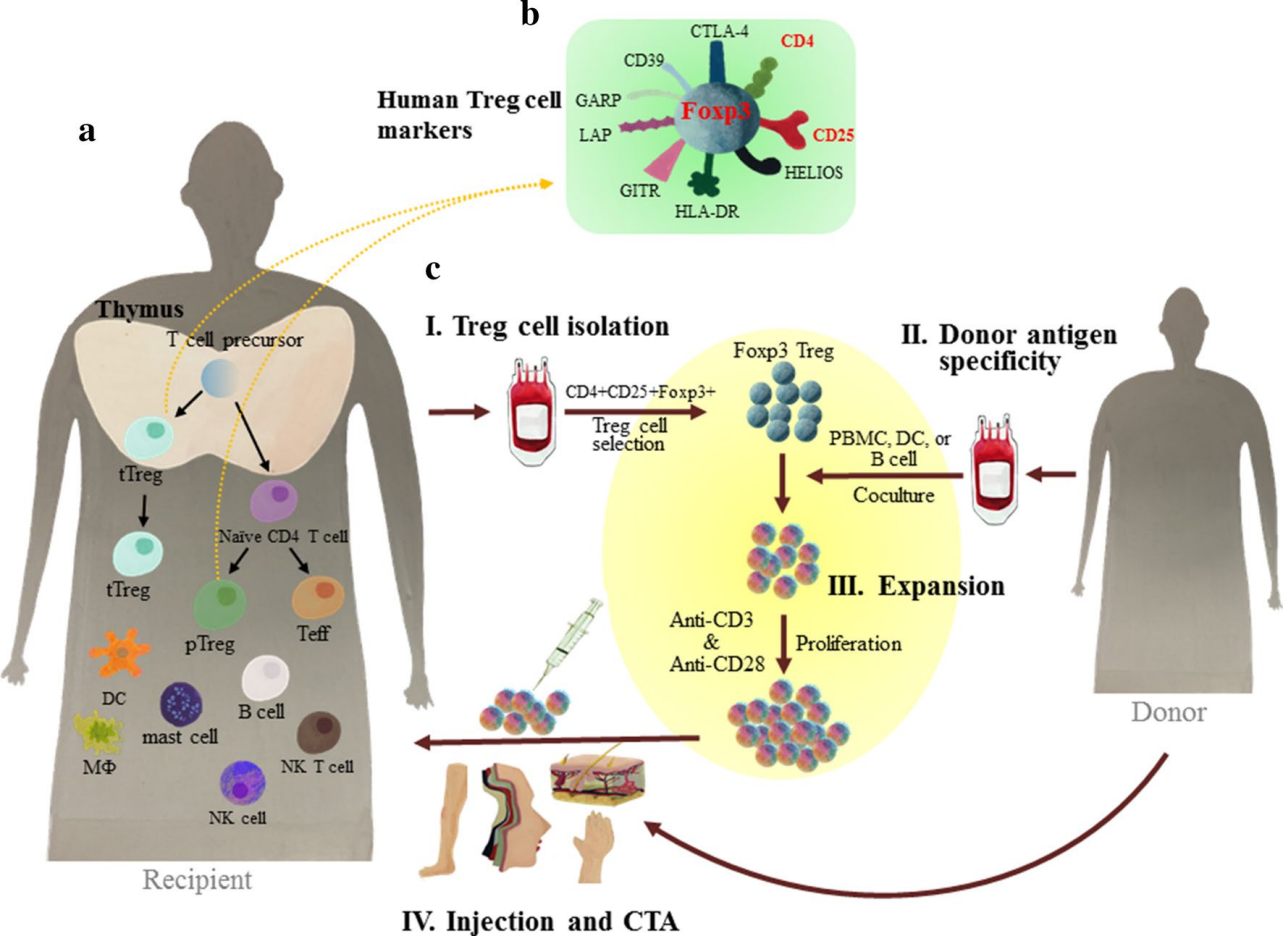
**a** Treg cells development pathways, **b** human Treg cell expressing markers, and **c** workflow of therapeutic application of donor antigen-specific Treg cells in CTA. **a** Treg cells development pathways. In the thymus, T cell precursors derived from bone marrow progenitor cells are developed into thymic Treg (tTreg) cells and exported to the periphery. Peripheral Treg (pTreg) cells are induced by naïve CD4^+^CD25^−^ T cells. **b** Human Treg cells expressing markers. Expression of CD4, CD25, FoxP3, CLTA-4, CD39, HLA-DR, LAP, GARP, and HELIOS is frequently observed in human Treg cells. **c** A workflow of therapeutic application of donor antigen-specific Treg cells in CTA. As a positive selection step to enrich CD4^+^CD25^+^Foxp3^+^ Treg cells, a sub-saturating concentration of anti-CD4 antibody and anti-CD25 antibody is used to capture CD4^+^ and CD25^bright^ fractions (I, isolation). To produce donor antigen-specific Treg cells, DCs (or APCs), B cells, or peripheral blood mononuclear cells (PBMCs) are applied (II, antigen-specificity). After priming in mixed lymphocyte reaction, Treg cells are stimulated with anti-CD3 antibody and anti-CD28 antibody for enrichment and expansion (III, expansion). Finally, expanded Treg cells are injected back to the recipient in CTA (IV, Treg cells injection and CTA)

So far, 322 clinical trials of Treg cell therapy have been listed on http://www.ClinicalTrials.gov registry at the time of writing this article (31-07-2017). However, none of them involves CTA. Nevertheless, current ongoing clinical trials of Treg cell therapy targeting induction of immune tolerance in organ transplantation and autoimmune disease may provide valuable lessons. Recent outcomes after phase I clinical trial of polyclonal Treg cells in patients with Type I diabetes mellitus (DM) have proved its safety [171]. Undergoing clinical trials of Treg cell therapy for allotransplantation and autoimmune diseases include kidney and liver transplantation, GVHD, Type I DM, Lupus, and other autoimmune-related diseases with polyclonal or donor antigen-specific Treg cells [160]. Given the superior efficacy of antigen-specific Treg cells with direct allospecificity in preclinical studies compared to polyclonal Treg cells, antigen-specific Treg cells have a favorable position in allotransplantation [164]. Regarding immune modulatory function, donor antigen-specific Treg cells have an advantage of immune site specific distribution against alloantigens and induction of immune tolerance. Thus, they could be, particularly, effective for CTA. In adoptive Treg cell transfer and immune tolerance, the following factors might contribute to graft acceptance: (1) time frame for infusion of Treg cells shortly before or at the time of transplantation or during a graft rejection episode, (2) mixed ratio of Treg cells to Teff cells for adoptive transfer of Treg cells in which Teff cells play a role in maintaining recipient’s immunity, and (3) co-treatment with immunosuppressive drugs, co-stimulatory molecule blockades, or/and cytokines. Indeed, the field of CTA is growing. Eliminating side effects caused by immunosuppressive drugs with Treg cells will nurture the field of allotransplantation in general. A schematic representation of Treg cells development pathways, human Treg cell expressing markers, and workflow of therapeutic application of antigen-specific Treg cells in CTA is shown in Fig. 1.

## Conclusions

CTA and organ transplantation share a common concept of alloimmunity. However, studies on CTA have revealed its unique characteristics different from organ transplantation as follows. First, rejection rate of hand or skin transplantation is exceptionally high. About 85–95% of patients experience transplantation rejection within a year, in sharp contrast to organ transplantation whose rejection rate is less than 10%. Second, CTA shows a tendency of resistance in spite of treatment with immunosuppressive drugs compared to organ transplantation which shows immune susceptibility. Third, CTA comprises multiple layers of tissues compared to organ transplantation which has rather homogeneous parenchyma. Fourth, monitoring transplantation immune rejection in CTA is relatively easy and visible.

Prevention of acute allorejection and long term survival of grafted tissues are two ultimate goals in CTA. Although the development and advancement of immunosuppressive drugs have changed the paradigm for allotransplantation and broke barriers for MHC class antigens in immune recognition, lifelong medication of non-antigen-specific immunosuppressive drugs and their side effects pose serious health issues. In this regard, application of Treg cells, particularly the generation of donor antigen-specific Treg cells to induce immune tolerance is an attractive task in allotransplantation of organs or tissues. Considering that CTA shows exceptionally high antigenicity, the strategy of inducing immune tolerance with Treg cells by controlling skin antigenicity seems desirable. Cumulative evidence has demonstrated its feasibility in preclinical studies. Currently, Treg cell therapy is being applied in clinical trials of organ transplantation and autoimmune diseases. However, clinical trial of Treg cell therapy has not been conducted in CTA yet. For adoptive transfer of Treg cells in therapeutics, issues concerning safety and quality control of functional Treg cells are beyond requirement. Particularly, expansion of GMP grade Treg cells with maximum purity that abundantly express Foxp3 without secreting pro-inflammatory cytokines is the first priority. Although Foxp3 is regarded as a gold standard for Treg cells expressing CD25, it has limitation because the expression of Foxp3 and CD25 can be easily altered in Tconv cells under inflammatory conditions. Identification of bona fide Treg cells expressing reliable markers is eagerly awaited for effective Treg cell therapy in allotransplantation. In addition, development of biomarkers to precisely measure the degree of engraftment and predict the progress of chronic rejection in a timely manner is essential for early intervention in CTA.

## Abbreviations

APCs: antigen presenting cells
CAR: chimeric antigen receptor
CCR4: c-c-motif chemokine receptor-4
CD: cluster of differentiation
C4d: complement 4d
CLA: cutaneous lymphocyte antigen
CTA: composite tissue allotransplantation
DCs: dendritic cells
DM: diabetes mellitus
Foxp3: forkhead box P3
GARP: garpin protein
GITR: glucocorticoid-induced tumor-necrosis factor (TNF)-receptor-related protein
GMP: good manufacturing product
GVHD: graft versus host disease
HLA: human leukocyte antigen
IL-2: interleukin-2
LAP: latency associated-peptide
MHC: major histocompatibility complex
mTOR: mammalian target of rapamycin
NFAT: nuclear factor of activated T cell
NF-kB: the nuclear factor-kappa B
NK: natural killer
PBMCs: peripheral blood mononuclear cells
NRP-1: neuropilin 1
PD-1: programmed death-1
RA: retinoic acid
STAT: signal transducer and activator of transcription
Tconv cell: conventional T cell
TCR: T cell receptor
TGF-β: transforming growth factor-β
Th1 cell: type 1 helper cell
Tr1 cell: type 1 regulatory T cell
T_EM_ cell: effector memory T cell
Treg cell: T regulatory cell
